# Lipopolysaccharide-Binding Protein (LBP) and Inflammatory Biomarkers in SARS-CoV-2 Hospitalized Patients

**DOI:** 10.3390/jcm14124075

**Published:** 2025-06-09

**Authors:** Aldanah Alshathri, Iman Bindayel, Wajude Alabdullatif, Ali Alhijji, Ahmed Albarrag

**Affiliations:** 1 Department of Community Health Sciences, College of Applied Medical Sciences, King Saud University, Riyadh 11433, Saudi Arabia; aldanah.alshathri@gmail.com (A.A.);; 2 Department of Internal Medicine, College of Medicine, King Saud University, Riyadh 11461, Saudi Arabia; 3 Department of Pathology, College of Medicine, King Saud University, Riyadh 11461, Saudi Arabia; aalbarrag@ksu.edu.sa

**Keywords:** SARS-CoV-2, inflammatory biomarkers, lipopolysaccharide-binding protein, cytokine storm, systemic inflammation, malnutrition, disease severity, NRS-2002

## Abstract

**Background/Objectives:** Infection with the severe acute respiratory syndrome coronavirus 2 (SARS-CoV-2) caused a global pandemic with far-reaching impacts on human activities. Moreover, direct viral damage and uncontrolled inflammation have been proposed as contributing factors to the severity of SARS-CoV-2 disease. Lipopolysaccharide binding protein (LBP) is also well recognized for its capability to trigger and modulate the host’s innate immune system by attaching to bacterial substances. Nevertheless, the pandemic has further emphasized the critical role of an effective host immune response in controlling viral infection and highlighted the detrimental effect of immune dysregulation. This study aimed to assess plasma levels of LBP and inflammatory biomarkers in SARS-CoV-2 patients with different malnutrition status and severity levels. **Methods**: This cross-sectional study was carried out in King Khalid University Hospital in Riyadh from December 2020 to December 2021. A total of 166 SARS-CoV-2 patients were recruited including 80 critical and 86 non-critical patients. Medical history, anthropometrical parameters, disease outcome information, and relevant biochemical parameters were extracted from medical records. Plasma samples were collected to test for LBP and inflammatory cytokines. Finally, nutritional risk was assessed by the Nutrition Risk Screening-2002 (NRS-2002) tool. **Results**: This cross-sectional study found no significant differences in LBP levels between critical and non-critical SARS-CoV-2 patients. However, LBP levels significantly correlated with IL-10, TNF-α and IL-6/IL-10 levels (Spearman’s rho = 0.430, 0.276 and −0.397 respectively; *p* < 0.001). **Conclusions**: This study confirmed the elevated inflammatory cytokines in hospitalized SARS-CoV-2 patients and their association with disease severity and malnutrition. These findings may support the mechanism of gut inflammation in order to develop new interventions that lower inflammatory biomarkers, disease severity, and aid in SARS-CoV-2 prevention and management.

## 1. Introduction

Newly emerging viral diseases have been a major public issue worldwide, negatively affecting people in several aspects [1]. Many viral illnesses have been documented in recent decades, including the severe acute respiratory syndrome coronavirus (SARS-CoV) in 2002 [2], H1N1 influenza in 2009 [3], the Middle East respiratory syndrome coronavirus (MERS-CoV) in 2012 [4], Ebola virus disease (EVD) in 2013 [5], and Zika virus in 2015 [6]. The most recent and active viral illness is the Severe Acute Respiratory Syndrome Coronavirus 2 (SARS-CoV-2), also known as the 2019 novel coronavirus (2019-nCoV) [7]. It was first reported on 31 December 2019, in Wuhan city, Hubei province, China [8]. On 30 January 2020, the World Health Organization (WHO) declared this outbreak an international public health emergency and soon afterward, on 11 March 2020, it was declared a global pandemic as it rapidly spread across the world [9]. Coronaviruses are proposed to have originated from bats [10] and these viruses have a positive-sense single-stranded RNA (+ssRNA) inside a spiked capsid that resembles the solar corona [8]. Compared to other positive RNA viruses, coronaviruses exhibit sophisticated mechanisms for attacking host cells, facilitated by their large genome. They can cross species barriers, infect humans, and commandeer host cells [8]. The spike (S) glycoprotein, responsible for the characteristic crown-like structure of coronaviruses, is essential for viral attachment and entry into host cells [11]. The S-protein comprises two subunits: S1 and S2, with the S1 subunit further divided into three domains: A, B, and C. The B domain enables SARS-CoV-2 and SARS-CoV to bind to the human angiotensin-converting enzyme-2 (hACE) receptor [12]. Additionally, genetic characteristics such as O-linked glycans, a polybasic furin cleavage site, and mutations in the S protein’s receptor-binding domain (RBD) enhance its interaction with hACE2 receptors, contributing to the virus’s high affinity and efficient spread [13]. The rapid spread of SARS-CoV-2, facilitated by its ability to transmit through respiratory and fecal–oral routes and evade host immune defenses has emphasized the need to understand its pathogenesis [14]. SARS-CoV-2 infection triggers excessive cytokine production, commonly referred to as a cytokine storm or cytokine release syndrome (CRS), which can lead to severe systemic inflammation and organ damage [15]. Pro-inflammatory cytokines such as IL-6 are elevated in critically ill patients, often indicating poor prognosis [16]. Previous reports have shown that individuals with obesity and malnutrition exhibit altered cytokine responses, including elevated IL-1β, IL-6, and TNF-α levels, which may worsen SARS-CoV-2 outcomes due to a heightened inflammatory state [17]. Recent studies suggest that bacterial lipopolysaccharide (LPS) may contribute to excessive immune activation during SARS-CoV-2 infection. LPS binding protein (LBP), a gut leakage biomarker, modulates the host’s immune response by binding to bacterial substances [18]. LBP has been widely studied in the context of critical illnesses such as sepsis and acute respiratory distress syndrome (ARDS), where higher levels have been associated with increased severity and poor clinical outcomes [17,19]. These findings support its relevance as a marker of systemic inflammation in the context of SARS-CoV-2. Disruption of the gut–blood barrier in severe SARS-CoV-2 cases may facilitate bacterial translocation, worsening systemic inflammation and disease severity [20]. In this context, the gut–lung axis has gained attention, where impaired intestinal barrier function and gut dysbiosis may drive systemic inflammation and pulmonary complications through translocation of endotoxins like LPS into circulation [21,22]. Malnutrition, including both undernutrition and obesity, has been linked to increased mortality and prolonged hospital stays in critically ill patients [23]. Both conditions have been associated with compromised gut integrity and immune function, which further increases the risk of microbial translocation and worsens inflammation [21]. Obesity-related pro-inflammatory states further hinder immune responses, increasing the risk of severe SARS-CoV-2 complications [24]. Patients with high BMI are at significantly greater risk of severe disease, ICU admission, and mortality [25]. Although several studies have explored the role of LBP in infection and chronic inflammation, data on its role in SARS-CoV-2 patients with varying nutritional status remain limited [26]. Building upon the previous work with the same cohort population [27], this study focuses on investigating plasma levels of LBP and its relation to inflammatory biomarkers in SARS-CoV-2 patients with varying malnutrition statuses, severity levels, and BMI categories, aiming to deepen the understanding of the relationship between inflammation, malnutrition, obesity, and disease outcomes.

## 2. Materials and Methods

### 2.1. Study Design and Participants

This cross-sectional study was conducted at King Saud University Medical City (KSUMC), Riyadh, Saudi Arabia, from January to June 2021. The study population comprised adult patients (≥18 years) who were admitted to the hospital with confirmed SARS-CoV-2 infection, as verified by reverse transcription polymerase chain reaction (RT-PCR). This study is a continuation of the research project previously published by Alabdullatif et al. (2023) [27]. Both studies used the same cohort, with identical recruitment procedures, sample handling, and analysis methods. However, the present work focused specifically on the plasma levels of lipopolysaccharide-binding protein (LBP) and its association to inflammatory cytokines namely tumor necrosis factor-alpha (TNF-α), interleukin-10 (IL-10), interleukin-6 (IL-6), and interleukin-8 (IL-8), and their associations with clinical outcomes. Patients were classified as critical or non-critical based on KSUMC’s intensive care unit (ICU) admission protocol. Exclusion criteria included pregnancy, lactation, ongoing chemotherapy, dialysis, malabsorptive disorders, or prior SARS-CoV-2 vaccination.

### 2.2. Ethical Approval

The study protocol was approved by the Institutional Review Board at King Saud University (approval number: E-20-5338), and all procedures were conducted in accordance with the Declaration of Helsinki. Informed consent was obtained from all participants or their legal representatives when direct consent was not possible.

### 2.3. Clinical and Demographic Data

Demographic and clinical data, including age, sex, body mass index (BMI), comorbidities, and vital signs at admission, were retrieved retrospectively from electronic health records. Anthropometric measurements were either collected upon admission or extracted from patient files. BMI was calculated using the standard formula (weight in kilograms divided by height in meters squared) and categorized according to CDC criteria [28]. For patients ≥ 65 years, modified BMI cutoffs were applied as per Porter Starr and Bales [29].

### 2.4. Blood Sampling and Laboratory Measurements

Venous blood samples (10 mL) were collected within 24–72 h after hospital admission. Samples were collected in EDTA tubes and centrifuged immediately at 1100–1300 rpm for 15 min to obtain plasma. Aliquots of plasma were stored at −80 °C until cytokine analysis. All samples were processed and stored at the KSUMC laboratory. Plasma concentrations of LBP, TNF-α, IL-8, IL-6 and IL-10 were quantified using enzyme-linked immunosorbent assay (ELISA) kits (e.g., MyBioSource, Cat. No. MBS268334). The assays were performed in accordance with the manufacturer’s instructions, and the detection range for each marker was included in the respective kit documentation. Quality control samples provided with the kits were included in each run to ensure assay reliability. All laboratory analyses were conducted by trained personnel who were blinded to the clinical status and outcomes of the participants to minimize bias. All assays employed the quantitative sandwich ELISA method and were performed in duplicate according to the manufacturer’s instructions. Absorbance was measured at 450 nm using a microplate reader, and cytokine concentrations were derived from a standard curve. The intra- and inter-assay coefficient of variation for all kits was below 10%.

### 2.5. Criteria for Determining Disease Severity

Patients were classified as critical based on the ICU admission protocol followed at King Saud University Medical City (KSUMC), as previously described in detail in the original publication [27]. This protocol prioritizes patients requiring life-saving interventions, including invasive mechanical ventilation, vasopressor support, or aggressive fluid resuscitation. Admission decisions were guided by objective clinical criteria such as marked abnormalities in vital signs, severe metabolic or respiratory disturbances, reduced urine output, significant hemoglobin decline, or signs of acute organ dysfunction. Patients who required hospital admission but did not meet these critical thresholds were categorized as non-critical.

### 2.6. Nutritional Assessment

Nutritional risk screening was performed at baseline using the Nutritional Risk Screening 2002 (NRS-2002) tool, recommended by ESPEN for hospitalized patients [30]. This assessment was conducted as part of the original study, where patients with a total score ≥ 3 were classified as at risk of malnutrition, according to established criteria. All assessments were performed by a single clinical dietitian who had prior experience with the tool and followed standardized procedures to ensure consistency [27].

### 2.7. Statistical Analysis

Statistical analyses were performed using SPSS version 26.0 (IBM Corp., Armonk, NY, USA). Data normality was assessed using Shapiro–Wilk tests. Continuous variables were expressed as mean ± standard deviation or median and interquartile range, as appropriate. Independent-samples *t*-tests or Mann–Whitney U tests were used to compare continuous variables between groups, while chi-square tests were applied for categorical variables. Correlation analyses were conducted using Pearson or Spearman tests, depending on data distribution. A *p*-value < 0.05 was considered statistically significant.

## 3. Results

### 3.1. Study Population

Out of 246 patients admitted with confirmed SARS-CoV-2 infection, 63 were excluded for not meeting inclusion criteria and 16 declined to participate. A total of 167 patients were enrolled in the study, including 81 critical and 86 non-critical cases.

### 3.2. Demographic, Anthropometric and Clinical Characteristics

**Figure 1:**
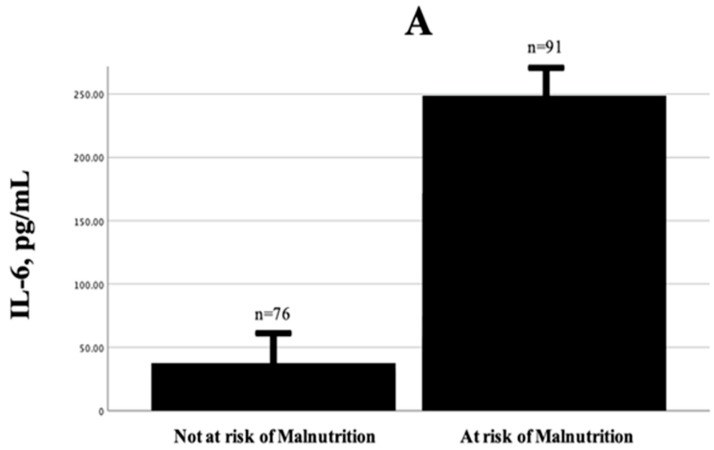

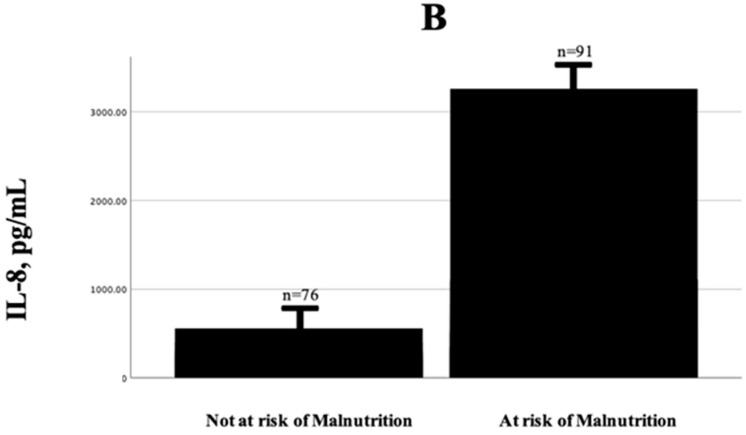
(**A**) Comparison of IL-6 levels between patients at risk of malnutrition and those not at risk, as classified by the NRS-2002. (**B**) Comparison of IL-8 levels between patients at risk of malnutrition and those not at risk, as classified by the NRS-2002.

Among the study population, 84 were female (50.3%) and 83 were male (49.7%). Critical patients were significantly older than non-critical patients (62.6 ± 15.9 vs. 57.6 ± 13.9 years, *p* = 0.031), but there was no significant difference in gender distribution (*p* = 0.396) Table 1. The overall mean BMI was 30.9 ± 7.2 kg/m^2^. No significant differences in BMI were observed between critical and non-critical groups (*p* = 0.447), and BMI categories were also similarly distributed (*p* = 0.361). Notably, 48.5% of participants had a BMI above 30 kg/m^2^ Table 1. Moreover, patients at risk of malnutrition (*n* = 91) showed markedly higher levels of IL-6 and IL-8 compared to those not at risk (*n* = 76), as shown in Figure 1A and B, respectively.

### 3.3. LBP and Its Relation to Inflammatory Biomarkers

No statistically significant differences were observed between critical and non-critical groups in LBP levels (*p* > 0.05). However, a positive moderate correlation was found between LBP and IL-10 (*p* < 0.001). Moreover, a positive, weak correlation was found between LBP and TNF-α (*p* < 0.001). Additionally, a negative, moderate correlation was found between LBP and IL-6/IL-10 (*p* < 0.001). Nevertheless, no correlation was found between LBP and other variables (*p* > 0.01) Table 2. Participants were stratified by BMI into three categories; IL-8 levels differed significantly among groups (*p* = 0.049), with the highest levels in those with BMI < 25 kg/m^2^. CRP levels were significantly higher in the BMI 25–30 kg/m^2^ group (*p* = 0.025). No significant differences were observed for LBP across BMI groups Table 3. Patients classified as at risk of malnutrition had no elevation in LBP, compared to not at-risk patients Table 4. Among the critical care group, non-survivors were significantly older than survivors (67.0 ± 16.8 vs. 59.6 ± 14.7 years, *p* = 0.032). However, no significant differences were found in BMI and LBP between survivors and non-survivors Table 5.

## 4. Discussion

This study explored the relationship between lipopolysaccharide-binding protein (LBP), inflammatory cytokines, and disease severity among hospitalized SARS-CoV-2 patients in Saudi Arabia. Although no statistically significant differences were observed in LBP levels between critical and non-critical cases, significant correlations were found between LBP and specific inflammatory markers, particularly IL-10 and TNF-α. These findings suggest a potential immunomodulatory role of LBP in the context of SARS-CoV-2, though not directly linked to disease severity in this cohort. LBP is a critical acute-phase protein involved in the host defense against Gram-negative bacteria by binding to lipopolysaccharides (LPS) and initiating immune signaling via the CD14-TLR4-MD2 complex [31]. Its elevation is generally interpreted as a marker of microbial translocation and systemic inflammation. While prior studies have demonstrated significantly elevated LBP levels in SARS-CoV-2 patients compared to healthy controls [32,33]. Our analysis did not reveal significant differences in LBP levels across disease severity categories or between survivors and non-survivors. These findings reflect differences in study design and comparison groups, and therefore are not directly comparable. This contrasts with a previous study that observed elevated LBP in ICU patients at diagnosis (T0) compared to non-ICU patients, though this difference diminished by day seven (T7) [34]. Such findings point out to a transient elevation of LBP early in critical illness, which may explain the lack of significant differences in our cohort if the timing of sampling post-infection varied. A novel aspect of our findings is the positive correlation between LBP and IL-10, a cytokine traditionally considered anti-inflammatory. This correlation aligns with the concept that LBP might be involved in regulating compensatory anti-inflammatory responses during acute viral infections [31]. Interestingly, we also identified a weaker, but significant, correlation between LBP and TNF-α, a pro-inflammatory cytokine that contributes to cytokine storm and tissue damage in severe SARS-CoV-2 [35]. Conversely, LBP was negatively correlated with the IL-6/IL-10 ratio, which may reflect a shift toward an anti-inflammatory profile in patients with elevated LBP. These findings suggest that LBP may function not merely as a marker of inflammation but also as a modulator of the inflammatory balance, echoing observations from the sepsis literature [36]. Despite a high prevalence of obesity in our cohort, BMI was not significantly associated with LBP levels or inflammatory cytokine ratios. This observation contrasts with reports linking obesity to increased LBP due to metabolic endotoxemia and altered gut permeability [37]. Notably, IL-8 levels were significantly higher in individuals with BMI < 25 kg/m^2^, a counterintuitive finding that may be attributable to age-related immune variation or unmeasured nutritional deficiencies [38]. Moreover, CRP levels were highest among participants with BMI 25–30 kg/m^2^, indicating possible low-grade inflammation in this subgroup, though without corresponding elevations in LBP or pro-inflammatory cytokines. Nutritional risk, evaluated using clinical assessment, did not correlate with elevated LBP levels. Although patients at risk of malnutrition were significantly older and more likely to be classified as critical, their LBP concentrations did not differ from those not at risk [36]. This further supports the notion that LBP levels may not reflect nutritional status in acute viral infections. Similarly, when analyzing outcomes within the critical care subgroup, we found that non-survivors were significantly older than survivors, consistent with prior reports on age as a risk factor for SARS-CoV-2 mortality [39]. However, no differences in BMI or LBP levels were detected between survivors and non-survivors. This study is the first in Saudi Arabia to evaluate LBP in the context of SARS-CoV-2 and its relation to immune response and disease severity. A key strength is the exclusion of confounding factors such as vaccination status, chronic immunosuppression, and conditions affecting protein metabolism. Additionally, biomarker analysis was conducted using a validated ELISA method, with samples collected within 24–72 h of admission and stored under standardized conditions, thereby reducing pre-analytical variability. Nonetheless, several limitations must be acknowledged. The absence of a healthy control group precludes definitive conclusions regarding baseline LBP elevations. Furthermore, cytokine and LBP levels were measured at a single time point, limiting our ability to track dynamic changes throughout the disease course. Funding constraints also restricted the scope of cytokine profiling and precluded inclusion of microbiome or endotoxin data, which could have enriched our understanding of LBP regulation in SARS-CoV-2. Despite these limitations, our findings contribute to addressing a regional gap in the literature by characterizing the inflammatory profile, including LBP, in hospitalized patients with SARS-CoV-2. This adds valuable insight for future studies exploring host–pathogen interactions and biomarker utility especially in Middle Eastern populations.

## 5. Conclusions

The SARS-CoV-2 pandemic has had significant effects on worldwide health and the global economy. SARS-CoV-2 pathogenesis and severity has been linked to LBP and inflammatory cytokines as they are considered essential components of the immune response. Although several studies including our research have found significant associations between SARS-CoV-2 and inflammation, the exact mechanism of inflammation and its effect on disease outcomes remains unclear. Hence, future studies are needed to illustrate and define the role of LBP and inflammatory cytokines in the pathogenesis and severity of SARS-CoV-2 among patients in different populations, at different ages, and with and without comorbidities. Moreover, the recognition of such markers will enable prioritizing of hospital resources and may allow the delivery of personalized treatments. Therefore, understanding how the severity of SARS-CoV-2 modifies the typical antiviral immune function and associated inflammatory markers is of great interest. To build on these findings, future studies should incorporate longitudinal sampling, broader cytokine panels, and healthy control groups to improve causal inference and enhance generalizability.

## Figures and Tables

**Table 1:** Baseline characteristics.

Characteristic	Total (*n* = 167)	Non-Critical (*n* = 86)	Critical (*n* = 81)	*p*-Value
Age (years)	59.54 ± 14.84	57.58 ± 13.90	62.62 ± 15.91	**0.031**
Female	84 (50.3%)	46 (53.5%)	38 (46.9%)	0.396
Male	83 (49.7%)	40 (46.5%)	43 (53.1%)	
Weight, kg	82.66 ± 19.58	82.11 ± 18.51	83.36 ± 20.48	0.678
BMI, kg/m^2^	30.87 ± 7.17	30.46 ± 0.67	31.31 ± 8.08	0.447
**BMI Categories**				
<25 kg/m^2^	34 (20.4%)	17 (19.8%)	17 (21%)	
25–≤30 kg/m^2^	52 (31.1%)	23 (26.7%)	29 (35.8%)	0.361
>30 kg/m^2^	81 (48.5%)	46 (53.5%)	35 (43.2%)	
**Inflammatory biomarkers**				
LBP, ng/mL	8.32 [5.19–10.1]	8.7 [5.5–10.7]	7.8 [5–9.7]	0.140
IL-6, pg/mL	24.63 [4.3]	23.93 [2.75]	26.80 [7.57]	**<0.001**
IL-8, pg/mL	71.01 [153.59]	32.47 [92.15]	124.55 [245.89]	**<0.001**
IL-10, pg/mL	266.23 [456.53]	206.76 [458.82]	248.76 [479.08]	0.674
TNF-α, pg/mL	131.17 [227.25]	124.92 [171.26]	151.88 [418.06]	0.125
IL-6/IL-10	0.1334 [0.23]	0.12 [0.21]	0.134 [0.24]	0.353
IL-10/TNF-α	1.73 [2.96]	1.85 [2.85]	1.53 [3.05]	0.315
CRP, mg/L	103.5 [110.6]	88.95 [112.5]	113.0 [120.13]	0.057

Note: Bold indicates statistically significant differences (*p* < 0.01).

**Table 2:** Correlation between LBP and inflammatory cytokines.

Variable	Correlation Coefficient	*p* Value
Age	0.102	0.188
Gender	-	0.970
BMI, kg/m^2^	−0.063	0.420
IL-6, pg/mL	0.009	0.906
IL-8, pg/mL	0.077	0.320
IL-10, pg/mL	0.427	**<0.001**
TNF-α, pg/mL	0.275	**<0.001**
IL-6/IL-10	−0.397	**<0.001**
IL-10/TNF-α	0.050	0.521
CRP, mg/L	−0.084	0.299

Note: Bold indicates correlation is significant at the 0.01 level (2-tailed).

**Table 3:** Differences in LBP and inflammatory cytokines based on BMI category.

Variable	BMI < 25 (*n* = 34)	BMI 25–<30 (*n* = 52)	BMI > 30 (*n* = 81)	*p*-Value
LBP, ng/mL	8.31 [5.01–11.1]	8.61 [5.4–10.6]	7.9 [5–9.7]	0.613
IL-6, pg/mL	26.1 [23.8–30.1]	23.9 [23.3–27.6]	24.63 [23.2–26.8]	0.154
IL-8, pg/mL	124.6 [63.04–316.6]	63.2 [1.75–181.9]	77.3 [17.1–155.34]	**0.049**
IL-10, pg/mL	200.44 [96.9–517.5]	316.7 [100.9–654.5]	212.1 [93.68–543.3]	0.384
TNF-α, pg/mL	127.3 [66–465.4]	138.65 [73.8–257.8]	130.6 [68.6–317.3]	0.922
IL-6/IL-10	0.15 [0.04–0.26]	0.1 [0.04–0.3]	0.12 [0.05–0.3]	0.398
IL-10/TNF-α	1.08 [0.5–3.3]	1.7 [1–4.4]	1.78 [0.7–4.2]	0.214
CRP, mg/L	115.5 [29.25–172.25]	129 [83.02–188.5]	85.25 [47.3–160.25]	**0.025**

Note: Bold indicates statistically significant differences (*p* < 0.05).

**Table 4:** Differences in laboratory tests and clinical outcomes in patients who were and were not at risk for malnutrition.

Variable	Not at Risk of Malnutrition	At Risk of Malnutrition	*p*-Value
Critical cases	8 (10.5%)	73 (80.2%)	**<0.001**
Non-critical cases	68 (89.5%)	18 (19.8%)	0.154
Age, years	52.8 ± 10.2	66.0 ± 15.9	**<0.001**
Female	37 (46.1%)	47 (51.6%)	0.73
Male	39 (51.3%)	44 (48.4%)	0.922
BMI, kg/m^2^	31.2 ± 6.3	30.6 ± 7.84	0.641
LBP, ng/mL	8.2 [5.3–9.6]	8.6 [5–10.7]	0.565
IL-6, pg/mL	23.93 [2.75]	26.10 [6.75]	**<0.001**
IL-8, pg/mL	32.5 [1.75–93.9]	99.3 [63.04–285.9]	**<0.001**
IL-10, pg/mL	186.3 [87.5–547.1]	266.9 [107.4–575.1]	0.144
TNF-α, pg/mL	117.9 [72.1–235.1]	148.2 [69.2–407.9]	0.243
IL-6/IL-10	0.14 [0.04–0.3]	0.11 [0.04–0.27]	0.836
IL-10/TNF-α	1.5 [0.75–3.6]	1.9 [0.6–4.05]	0.928
CRP, mg/L	86 [46.7–162]	111.5 [61.37–179]	0.169

Note: Bold indicates statistically significant differences (*p* < 0.01).

**Table 5:** Differences in laboratory tests and clinical outcomes in critical survivors and non-survivors.

Variable	Survivors (*n* = 49)	Non-Survivors (*n* = 33)	*p*-Value
Age, years	59.58 ± 14.71	67.03 ± 16.77	**0.032**
Female	20 (41.7%)	18 (54.5%)	0.254
Male	28 (58.3%)	15 (45.5%)	
BMI, kg/m^2^	31.2 ± 6.3	30.6 ± 7.84	0.641
<25 kg/m^2^	9 (18.4%)	9 (27.7%)	0.400
25–30 kg/m^2^	20 (40.8%)	9 (27.7%)	
>30 kg/m^2^	20 (40.8%)	15 (45.5%)	
LBP, ng/mL	7 [4.7–9.5]	8.46 [5.94–9.85]	0.128
IL-6, pg/mL	25.3 [23.3–29.9]	28.3 [23.9–36.2]	**0.035**
IL-8, pg/mL	93.9 [32.5–278.2]	155.3 [81.3–365.1]	0.102
IL-10, pg/mL	248.75 [98.1–577.2]	239.3 [102.5–556.0]	0.974
TNF-α, pg/mL	110.8 [60.4–252.63]	260.45 [83.3–659.8]	0.102
IL-6/IL-10	0.13 [0.04–0.3]	0.1 [0.07–0.3]	0.681
IL-10/TNF-α	1.9 [0.9–4.7]	1.2 [0.4–2.5]	0.061
CRP, mg/L	144.5 [69.4–188.5]	98 [61.4–162.5]	0.10

Note: Bold indicates statistically significant differences (*p* < 0.05).

## Data Availability

The data presented in this study are available only upon request from the corresponding author.
